# Synergy in Neuroimaging: PET-CT and MRI Fusion for Enhanced Characterization of Brain Pathology

**DOI:** 10.7759/cureus.74353

**Published:** 2024-11-24

**Authors:** Nivedita Radder, Sameer Sonar, Avinash Nanivadekar, Shrinivas Radder

**Affiliations:** 1 Diagnostic Radiology, University of Arkansas for Medical Sciences, Little Rock, USA; 2 Nuclear Medicine/PET-CT, Ruby Hall Clinic, Pune, IND; 3 Diagnostic Radiology, Canon Medical Systems Corporation, Tokyo, JPN

**Keywords:** brain fdg pet, brain pathology, brain tumors (primary or brain metastasis), pet ct scan, pet-mri, pet-mri fusion

## Abstract

Background: Accurate diagnosis and understanding of brain disorders are crucial for the best treatment. While multimodal neuroimaging is essential, it has its limitations. Conventional computed tomography (CT) and magnetic resonance imaging (MRI) provide detailed anatomical information but lack molecular insights, while 18F-fluorodeoxyglucose-positron emission tomography (FDG PET) offers metabolic data but often has limited spatial resolution.

Objective: This study aimed to assess the potential of combining 18F-FDG PET with MRI to characterize brain disorders compared to standard imaging methods.

Methods: Fifty patients suspected of having brain tumors underwent 18F-FDG PET-CT and then MRI (including 3D fluid-attenuated inversion recovery (FLAIR)) after CT scans revealed suspicious lesions. The images were combined and PET-MRI findings were compared to the initial CT interpretations.

Results: The combination of PET-MRI significantly improved diagnostic accuracy in 20 out of the 50 patients (40%). Importantly, it identified and characterized brain lesions missed by CT in two patients (4%). In patients with known dementia or epilepsy, PET-MRI revealed specific metabolic patterns in affected brain areas.

Conclusion: 18F-FDG PET-MRI fusion shows greater sensitivity and specificity than standard imaging techniques for various brain disorders. It provides valuable insights into structural and functional abnormalities, potentially leading to improved diagnosis, treatment planning, and patient outcomes.

## Introduction

Neuroimaging is a cornerstone in the management of brain pathologies. Magnetic resonance imaging (MRI) is a key technique in the field due to its highly detailed anatomical information; however, it cannot characterize the metabolic and physiologic components that often underlie pathologies. Imaging at the molecular level by positron emission tomography (PET) fills this gap because it provides information about metabolic activity and, thus, about essential biomolecular processes. With the arrival of hybrid PET-MRI systems, a significant improvement has been achieved that unifies two powerful diagnostic tools and provides an overall approach to brain pathologies [1]. Allowing structural, functional, and molecular information to be obtained simultaneously offers the potential of a comprehensive lesion characterization that will aid in selecting patients appropriate for targeted therapy [2]. The clinical potential of PET-MRI is high, yet the worldwide dispersion and cost remain limiting factors. In this article, existing practical uses of positron emission tomography-computed tomography (PET-CT) and limited sequence MRI fusion are explored with emphasis on patient-specific gains and their broader applicability in clinical practice.

Role of PET-CT-MRI fusion in neuroimaging diagnostics

This is where a fusion of PET and MRI data can offer complementary information to what either technique alone provides. The combination of these two strategies, which is known as ‘anatomo-pathophysiological’, allows a human-like view of the disease processes considering its anatomical and molecular aspects together [1].

Improved brain pathology characterization

The fusion of PET data, reflecting metabolic activity, with high-resolution anatomical MRI images significantly enhances the characterization of brain pathologies. This is particularly valuable in oncology, where it aids in differentiating between tumor recurrence and treatment-related changes, a distinction often challenging with conventional imaging [3].

Better treatment planning and monitoring

Fusion of PET-MRI has a very important role in radiation treatment planning by identifying regions with metabolism activity accurately [4]. This focused targeting limits collateral damage to healthy tissue and increases the efficacy of the treatment. Additionally, real-time feedback on treatment response provides an opportunity for individualized dose adjustments to maximize the therapeutic benefit.

Reduced radiation exposure and enhanced patient comfort

PET-MRI fusion serves as a 'one-stop-shop' imaging method. It cuts down the need for several scans, which lowers radiation exposure [5]. Also, since we use one MRI 3D fluid-attenuated inversion recovery (FLAIR) sequence, the quicker scan times make patients more at ease and willing to cooperate.

## Materials and methods

This retrospective study was conducted at a tertiary care center and involved a cohort of 50 patients who underwent PET-CT scans due to concerns regarding potential brain lesions. Most participants had a significant medical history, primarily related to cancer, or were under evaluation for possible epilepsy-related dementia. The study specifically targeted patients aged 40-80 years of all genders who presented with brain masses, had a documented history of epileptic seizures and migraines, or displayed neurological symptoms indicative of dementia.

To refine our analysis, we excluded patients from the study who could not undergo MRI due to contraindications, such as specific implanted devices or severe claustrophobia, and those who could not complete the MRI procedure for any other reasons. This exclusion criterion ensured that our analysis focused solely on patients compatible with the required imaging protocols.

The evaluation was comprised of PET-CT and single-sequence MRI imaging technologies. The PET-CT scans were performed using a GE Discovery 16 STE scanner (GE Healthcare, USA), renowned for its high sensitivity and specificity in detecting metabolic processes. In the PET imaging protocol, we administered 10 millicuries of 18F-fluorodeoxyglucose (18F-FDG). Patients underwent a standard uptake period of 40 minutes before the dynamic emission scan, which lasted for the initial seven minutes and was conducted using three-dimensional acquisition techniques. This approach ensured robust metabolic data collection and enhanced image quality.

For the MRI component, we utilized the Philips Achieva 1.5 Tesla scanner (Philips Healthcare, Amsterdam, the Netherlands), which offers superior resolution and contrast for brain imaging. The MRI sequences primarily employed were FLAIR sequences, which are highly effective in identifying cerebral edema and lesions.

Furthermore, image fusion techniques were employed to create co-registered PET and MRI brain images. This fusion was performed using AW Cortex ID software (GE Healthcare, USA), which facilitated the integration of the metabolic information from PET with the anatomical data from MRI. Through this process, we generated Cortex ID maps that depicted metabolic alterations, allowing for detailed visualization of abnormal functions within the brain. The localization of these pathological areas was meticulously analyzed using the co-registered images and maps, providing insights into the underlying metabolic and functional changes associated with the patient's conditions.

## Results

The authors conducted PET-MRI fusion imaging in 50 patients. The most common diagnosis from scanning the brain was as follows: malignant metastatic intracranial masses (65%, 30/50); benign mass, n=5; dementia, n=5; epilepsy, n=3; Parkinson's disease, n=2; and no significant abnormality found in patients with type 1 diabetes mellitus (DM), n=5.

Brain tumors

**Figure 1:**
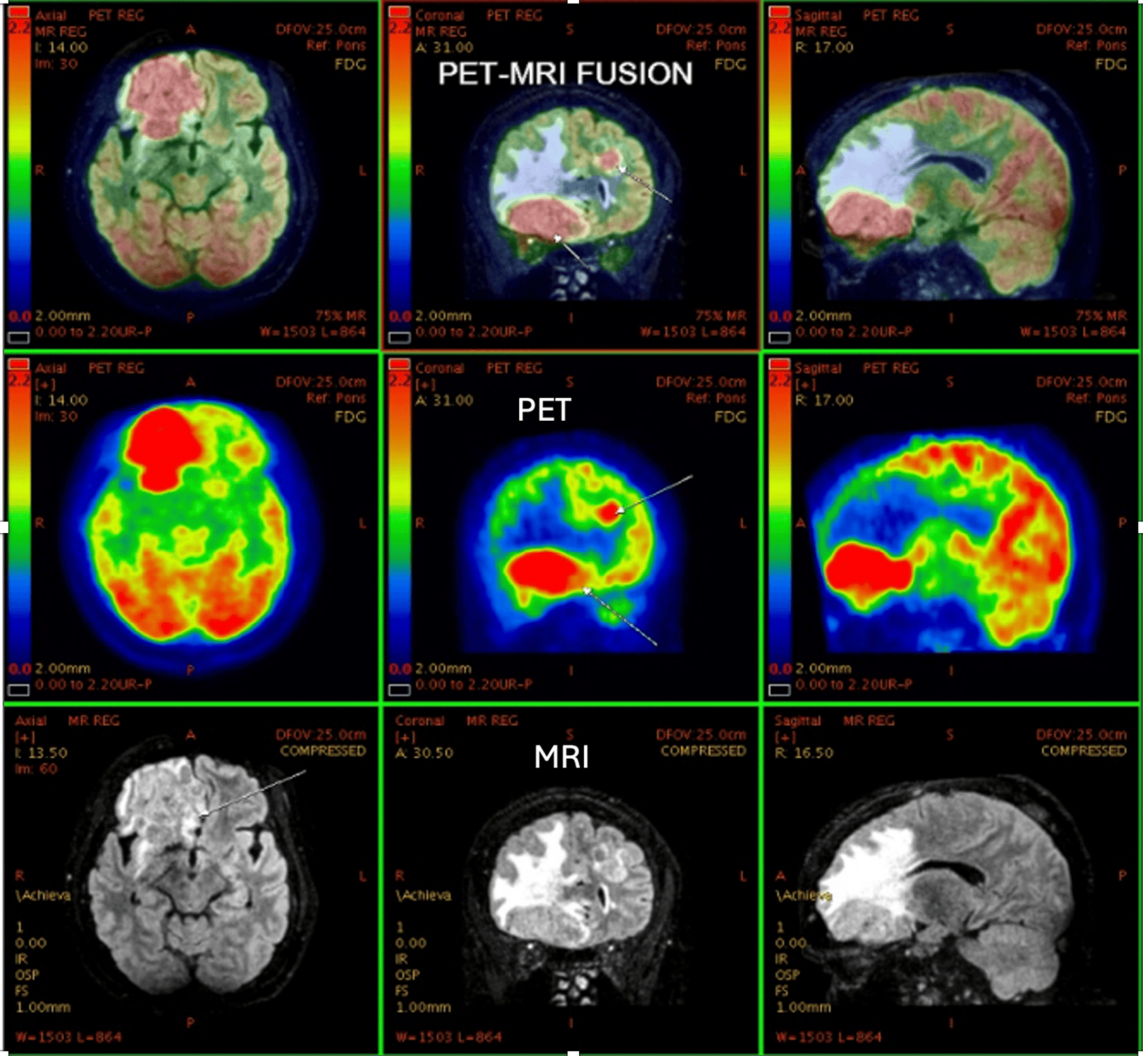
58-year-old female, known case of Ca right breast. Post-operative and chemotherapy status. Presented with complaints of left shoulder pain and lower backache for 15-20 days. CT shows an ill-defined heterogeneous area in the right frontal region. PET-MRI brain study shows heterogeneous large metabolically active lesions in the right basifrontal and left frontal regions with perilesional edema and mass effect, suggesting metastatic lesion. PET: positron emission tomography; MRI: magnetic resonance imaging; CT: computed tomography

**Figure 2:**
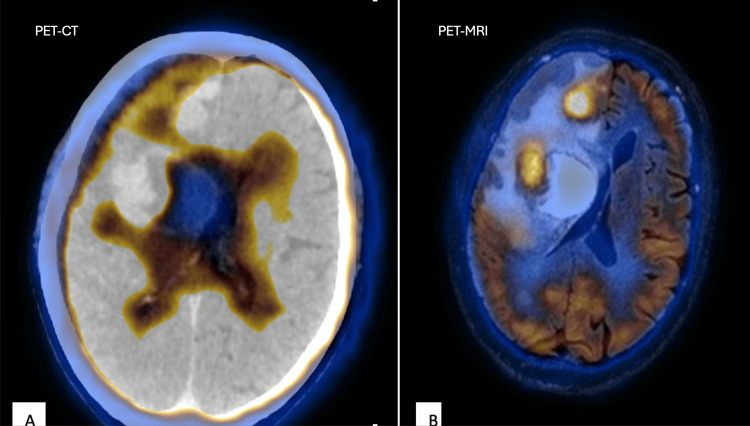
41-year-old female with a history of episodes of unconsciousness. (A) PET-CT shows an intense metabolically active ill-defined lesion in the right frontal region. (B) PET-MRI fusion was done to characterize the lesion. It shows a large solid cystic lesion in the right frontal region with surrounding edema and intense FDG uptake in the solid component, suggesting a primary neoplastic etiology. PET: positron emission tomography; MRI: magnetic resonance imaging; CT: computed tomography; FDG: fluorodeoxyglucose

**Figure 3:**
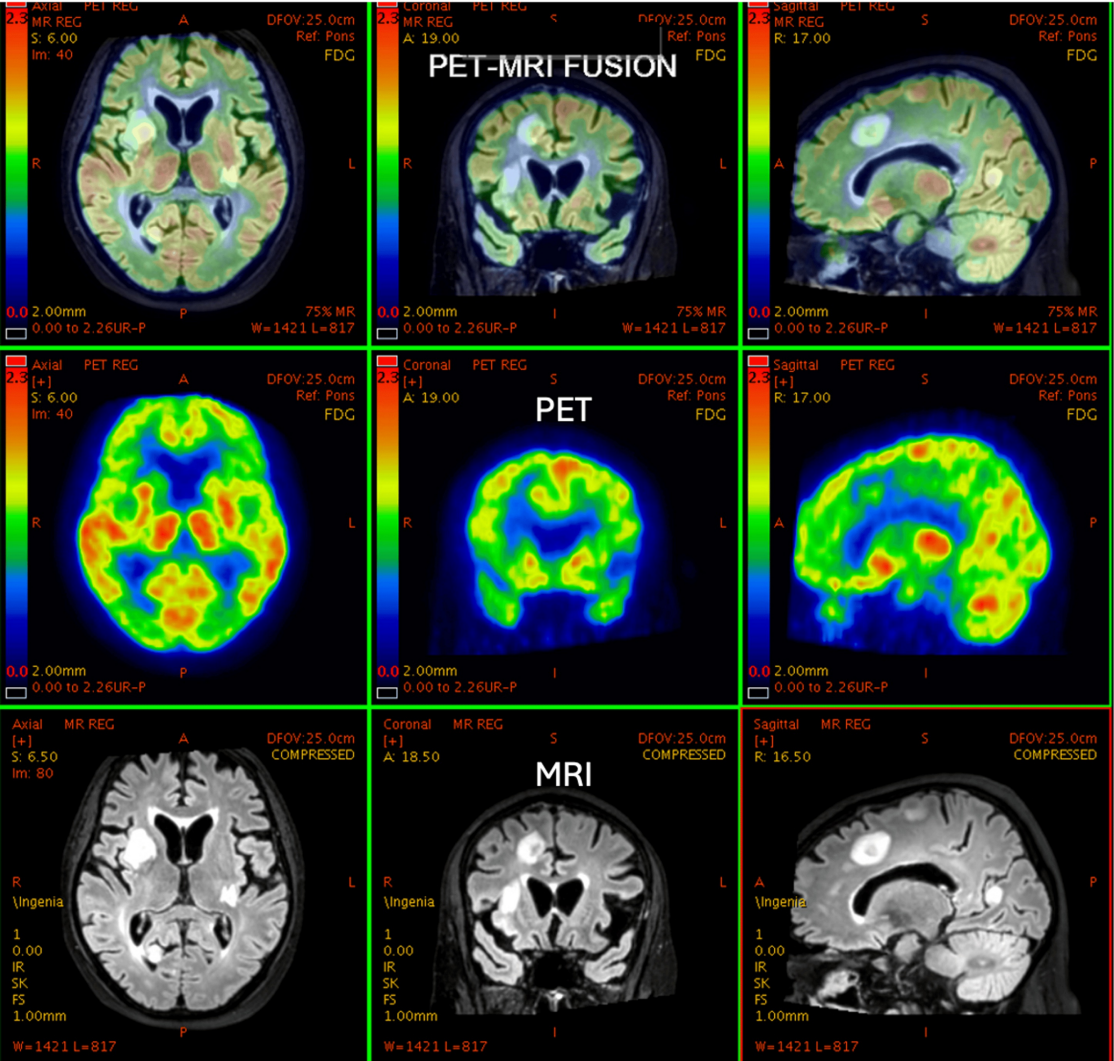
51-year-old male, K/c/o Ca prostate with brain metastasis. Post-radiotherapy to the brain. PET-MRI shows a reduction in metabolic activity of metastatic brain lesions, suggesting a response to therapy. PET: positron emission tomography; MRI: magnetic resonance imaging

**Figure 4:**
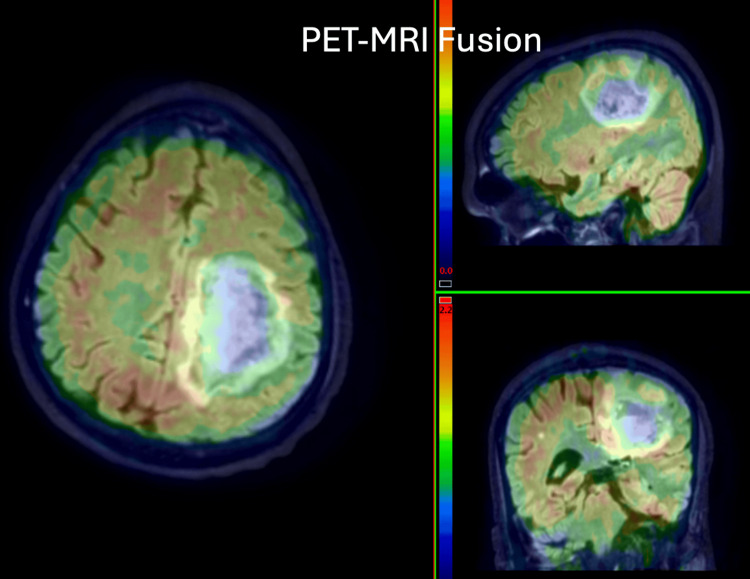
46-year-old male with a known operated case of high-grade lymphoma at D6-D7 level. MRI is suggestive of SOL in the left parietal region. PET-MRI shows hypometabolism within the lesion. Histopathology revealed low-grade glioma. PET: positron emission tomography; MRI: magnetic resonance imaging; SOL: space-occupying lesion

**Figure 5:**
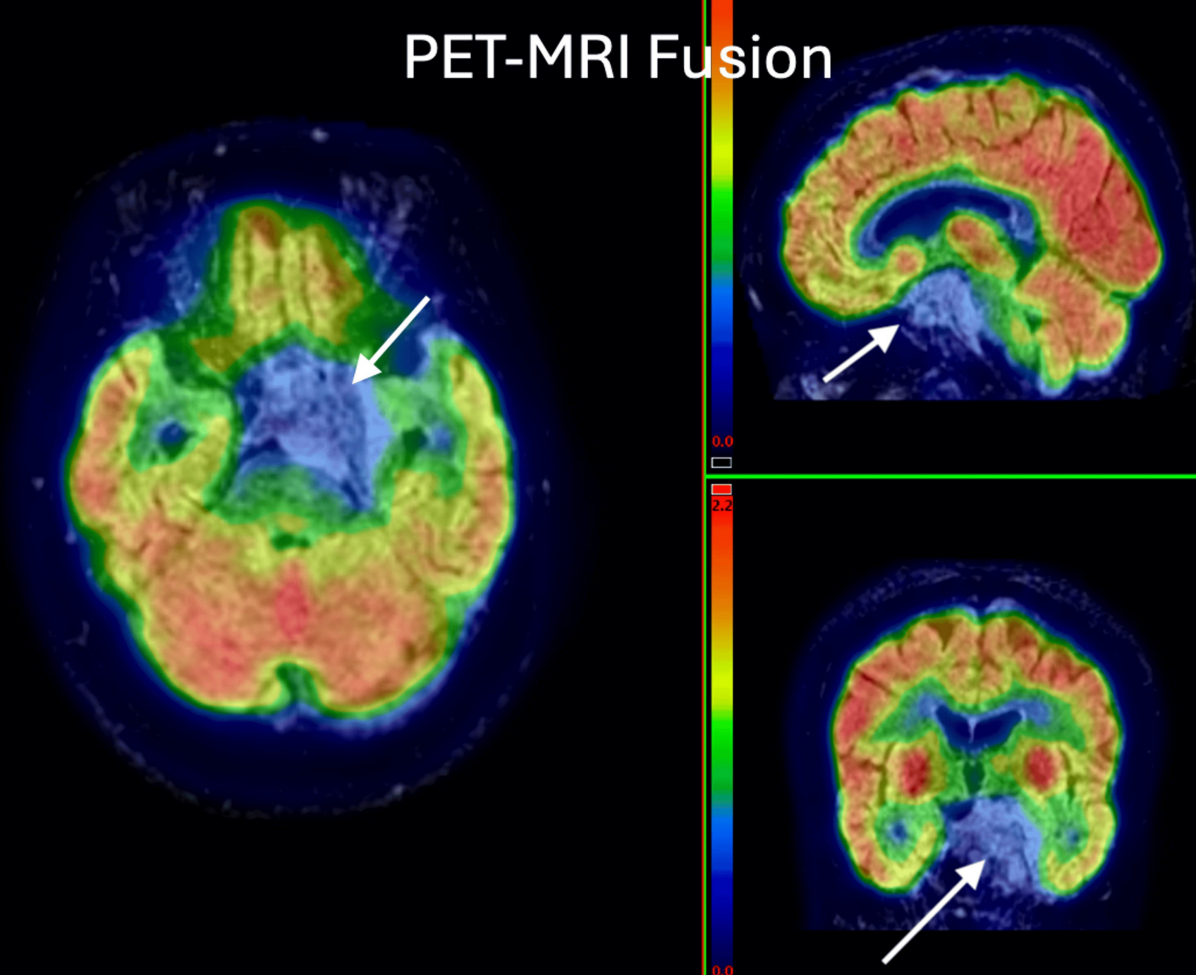
71-year-old female with a history of imbalance while walking and visual disturbance. PET-MRI brain study shows a large pituitary macroadenoma with no FDG activity. PET: positron emission tomography; MRI: magnetic resonance imaging; FDG: fluorodeoxyglucose

The fusion of PET and MRI was found to be useful in the attempt at the characterization of brain tumors. PET-CT alone can be confusing because of high background brain metabolism, but the combination with high-resolution MRI made it easier to differentiate benign from malignant lesions. This technique allowed identification at the cellular level of viable tumors as opposed to radiation-induced necrosis (Figures 1-5).

Dementia

PET-MRI fusion provided valuable insights into various types of dementia:

Alzheimer's Disease

**Figure 6:**
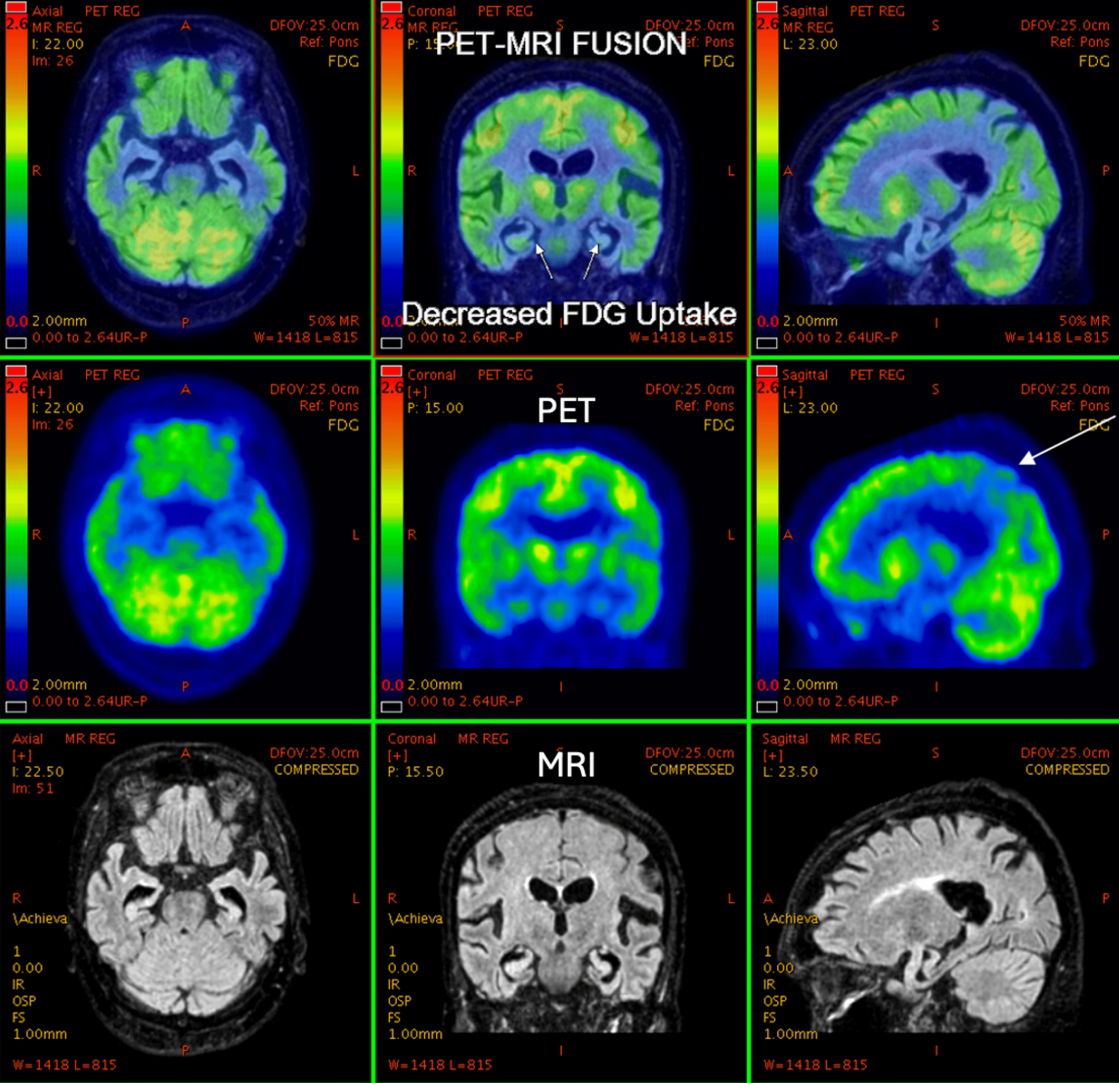
68-year-old female with a history of dementia, suspiciousness, and imbalance while doing day-to-day work for two years. Symptoms worsened for two months. PET-MRI brain study shows bilateral temporal lobes (predominantly mesial temporal lobe) and bilateral parietal lobes. Posterior cingulate gyrus shows diffuse atrophy and a symmetrical significant reduction in FDG uptake, suggesting temporoparietal pattern dementia - likely to be Alzheimer’s disease. PET: positron emission tomography; MRI: magnetic resonance imaging; FDG: fluorodeoxyglucose

**Figure 7:**
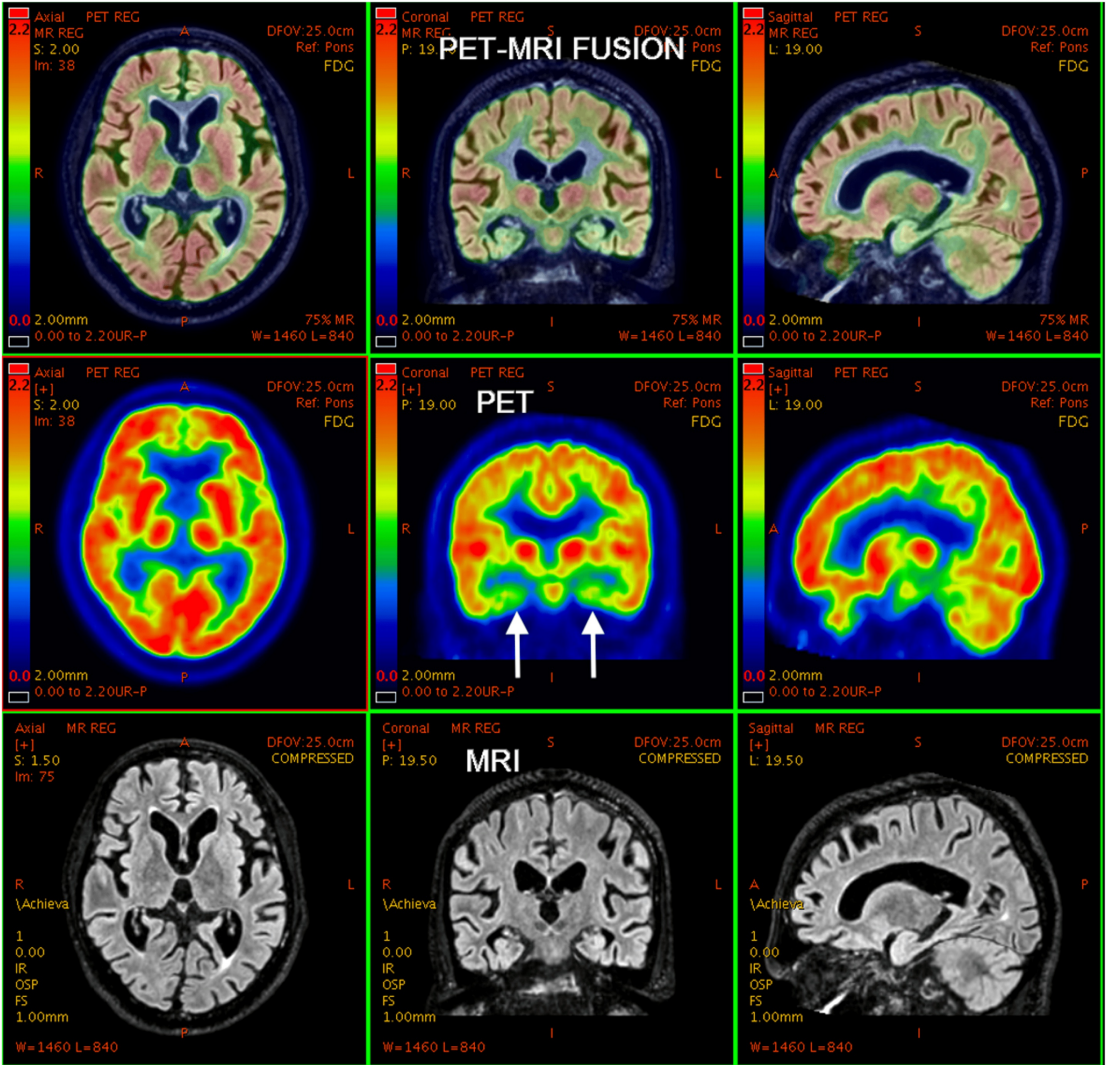
54-year-old male with progressive imbalance, forgetfulness, and dysarthria for one year. FDG uptake shows a homogeneous and symmetrical pattern in bilateral basal ganglia, frontal eye fields, posterior cingulate cortex, and visual cortex. Mild, relatively lower metabolic activity is observed in the medial temporal cortex. No areas of hypometabolism are noted. No metabolic alterations are seen. PET: positron emission tomography; MRI: magnetic resonance imaging; FDG: fluorodeoxyglucose

The posterior cingulate gyrus is a characteristic region of early Alzheimer's disease hypometabolism and was strikingly seen (Figures 6-7) [6].

*Dementia With Lewy Bodies* 

Patterns of hypometabolism mainly in the occipital cortex and visual association cortices with relative retention at the posterior cingulate gyrus and mesial temporal lobe were noted [6].

Frontotemporal Dementia

As predicted, hypometabolism was centered in the frontal and temporal lobes.

Seizure disorder

**Figure 8:**
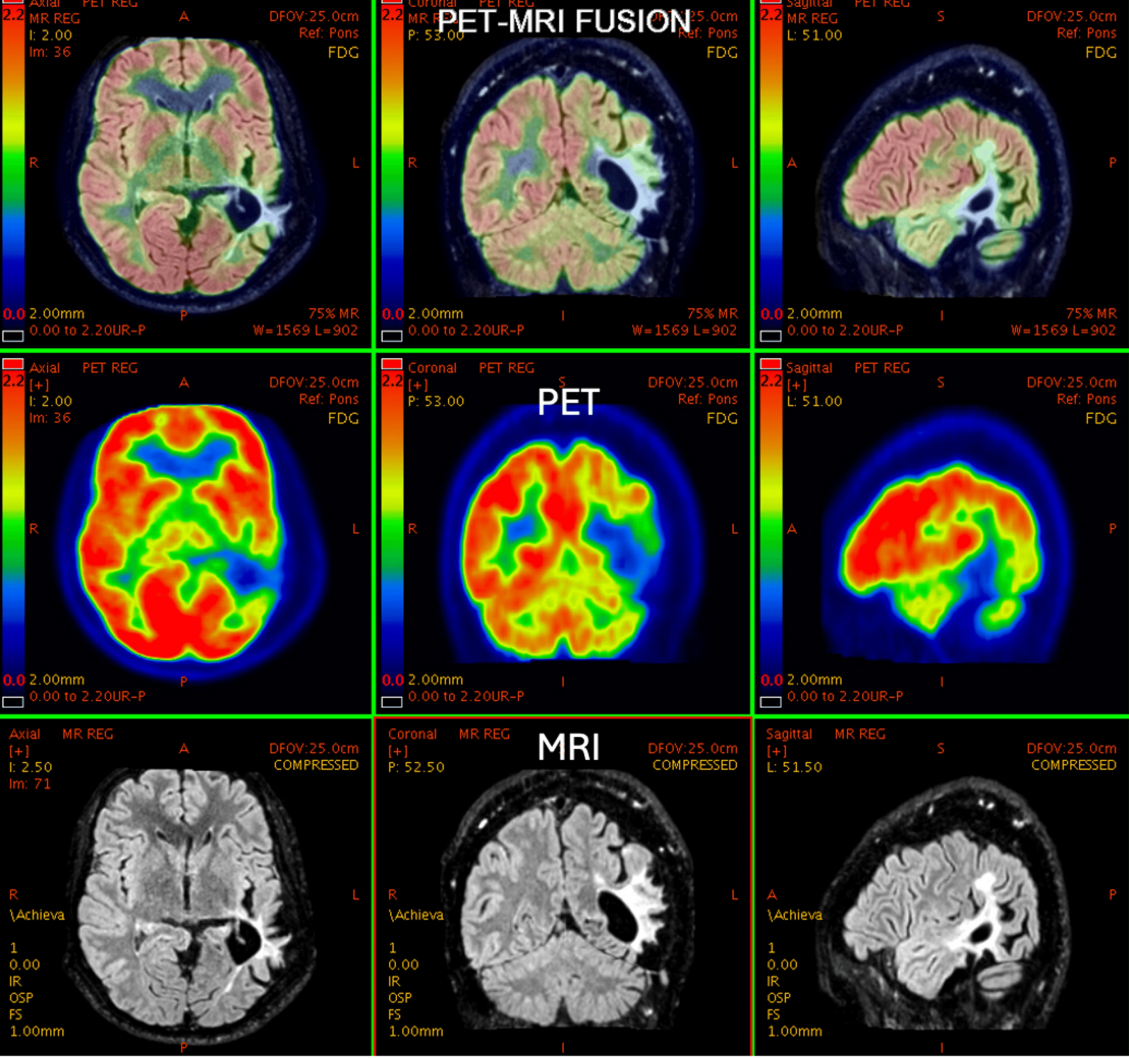
25-year-old male with a history of seizure disorder. PET-MRI brain study shows a gliotic area involving left parietal region, left superior temporal gyrus, and left middle temporal gyrus with prominence of overlying sulcal spaces underlying left temporal horn - left lateral ventricle confluence. Corresponding areas of PET images show decreased FDG uptake compared to the opposite side, suggesting decreased metabolic activity secondary to gliosis and volume loss. PET: positron emission tomography; MRI: magnetic resonance imaging; FDG: fluorodeoxyglucose

**Figure 9:**
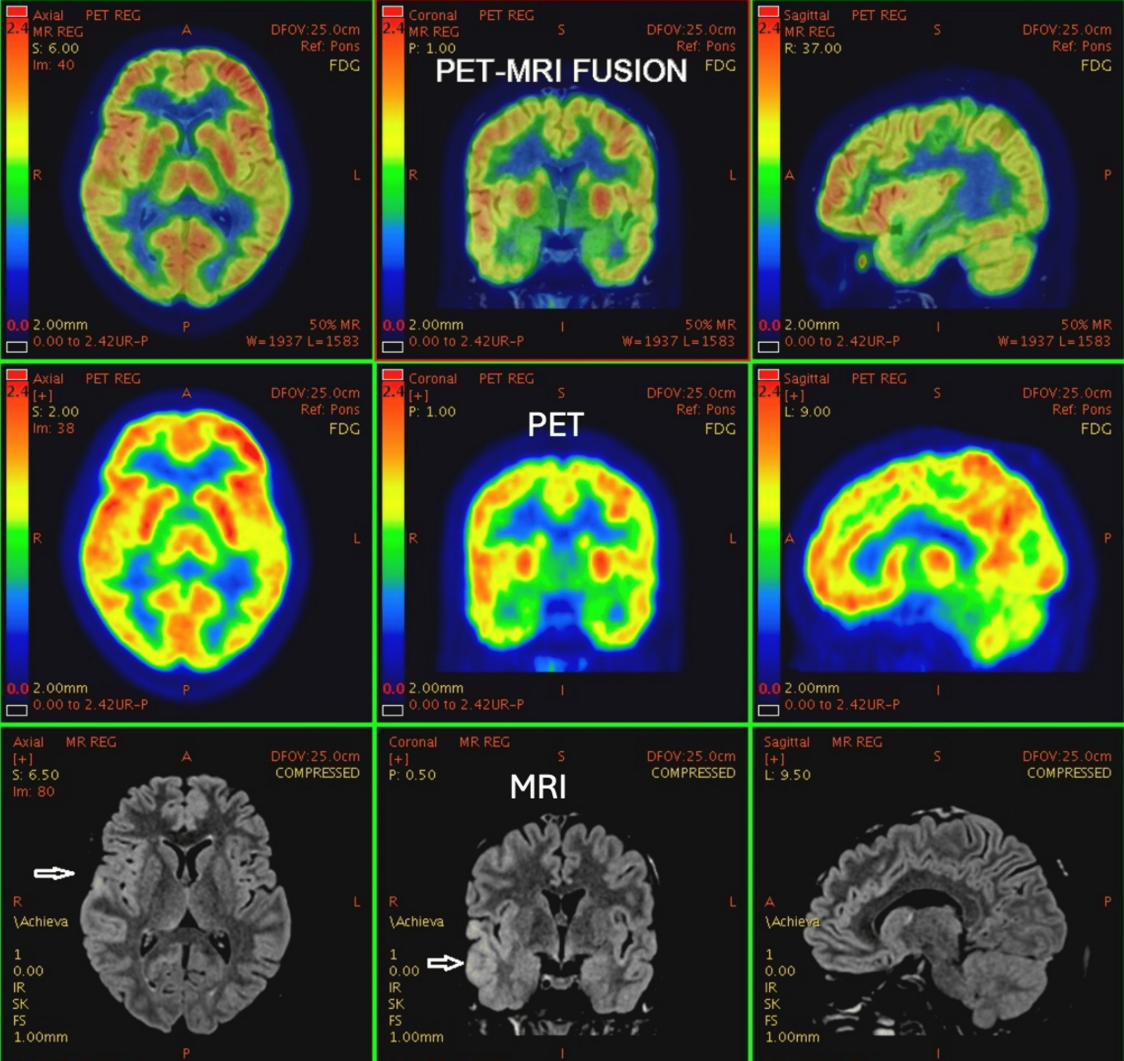
27-year-old female, K/c/o seizure disorder on treatment. C/o staring looks, irrelevant talk, bradycardia, and headache for 7-10 days. PET-MRI study shows the increased signal on FLAIR image in the right lateral temporal lobe and right insular cortex with a mild increase in FDG activity - likely to be postictal edema, although similar appearance could be seen in misalignment. PET: positron emission tomography; MRI: magnetic resonance imaging; FDG: fluorodeoxyglucose; FLAIR: 3D fluid-attenuated inversion recovery

The fusion of PET-MRI facilitated the co-localization of anatomical and metabolic abnormalities in patients with seizure disorders, offering complementary diagnostic information. The epileptogenic brain areas showed reduced FDG uptake, indicating that the metabolic activity in these regions had decreased (Figures 8-9).

Migraine

**Figure 10:**
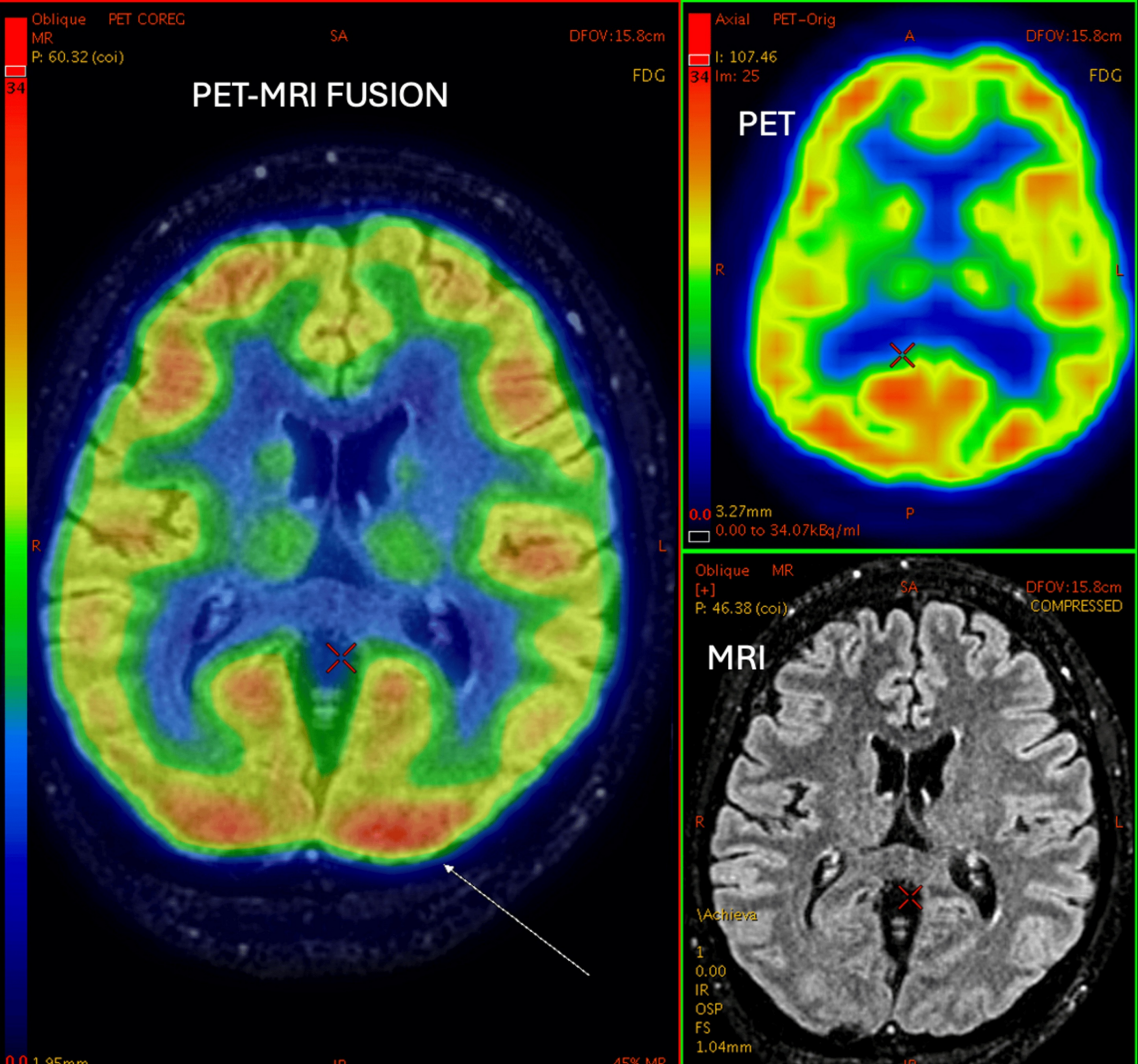
58-year-old male, K/c/o migraine. C/o imbalance, raised morning BP, mild morning headache, and fatigue for 10 days. PET-MRI brain study shows the primary visual cortex on the left side with increased metabolic activity (>2.3 SD). No underlying structural abnormality is seen. PET: positron emission tomography; MRI: magnetic resonance imaging; BP: blood pressure

We found different PET-MRI fusion-based changes in brain metabolism for migraine patients. On one hand, migraine attack-related increased activation of the vestibulo-thalamo-cortical pathway was seen, while on the other hand, significant hypometabolism in pain-processing regions (insula, anterior cingulate cortex (ACC), posterior parietal cortex (PPC)) at rest points to a common primary metabolic dysfunction underlying both hemodynamic changes associated with attacks as well as background processes (Figure 10). 

## Discussion

PET-MRI fusion studies reveal that the method effectively depicts what happens in the context of some diseases in the brain. It can be said as if the picture becomes clearer than in situations where PET-CT or MRI has been done alone [7-18].

Why this hybrid imaging is so exciting?

Spotting Trouble Early

Therefore, when the metabolic activity obtained from PET scans and the detailed anatomical data from MRI are brought in, one can detect even the slightest of issues with the brain. It is beneficial when conventional modalities only give us uncertainty.

Diagnosing With Confidence

This is because PET-MRI fusion offers a better opportunity to provide correct diagnoses of several neurological disorders. Whether boys are susceptible to a brain tumor, a diffuse cerebral disease, dementia, or epilepsy, having such metabolical and anatomical data guides the right decision.

Targeting Treatment With Precision

It is crucial to note the accuracy of every treatment session for brain tumors. PET-MRI fusion allows us, thus, to delineate the tumor margins accurately, which is mandatory for surgery planning or radiation therapy.

Telling the Difference

On other occasions, it becomes challenging to differentiate whether a tumor is still growing or exhibiting features that may have resulted from previous treatment. PET-MRI combination enables us to distinguish between the two, which leads us to the right strategy for managing the case.

The reason behind using both MRI and CT alone is that they could be more efficient.

In many cases, an MRI or CT scan, which are excellent imaging studies, will show us the brain and its pathology but will not depict the early changes in metabolism, which might suggest a problem. Think of it like this: before the smoke even rises and the smoke forms, it is sometimes as though the brain's engine is stalled.

Some specific limitations

Missing Early Warning Signs

Most neurological disorders develop from minor modifications in metabolism that would not normally be detected by routine imaging. Even when the structural change is clear, it could be too early for the most radical treatments, including dementia.

Too Much Overlap

The reason is that many of the brain conditions appear to be almost identical in their MRI or CT results; hence, their diagnosis is very complicated. It is like making an attempt to distinguish a bird by the silhouette that it casts.

Subtle Changes, Big Impact

It is possible to subdivide the brain conditions citing that some progress very slowly or include highly minimum alterations, which cannot be detected through MRI scans with great detail. This means that many people may receive a diagnosis later than they should and, therefore, will be inadequately treated [1].

## Conclusions

This work re-emphasizes the benefits of the combined use of imaging techniques in the diagnosis and management of brain disorders. Here, we have also shown the utility of using 18F-FDG PET with MRI in identifying and differentiating the pathologies in the brain that cannot be identified adequately by MRI or PET alone. Our work's other, more novel message is the value of a combined anatomical-functional-molecular imaging model. This wide-ranging perspective is unexpectedly helpful, especially in situations like tumors, epilepsy, and dementia, where other approaches might prove to be inadequate. The normal tissue may be exploited to check for structural abnormalities and metabolic function, hence offering a better insight that significantly impacts the patient's course of action. By providing evidence that PET-MRI fusion works, this paper’s scientific value is in demonstrating the need for this technique to be adopted by more hospital facilities. These results support the need for an anatomo-pathophysiological model, whereby information from structural imaging is combined with metabolic details to make more precise clinical decisions. Besides providing earlier and more accurate diagnoses with subsequent better treatment approaches, this strategy also improves patient care. In addition, PET-MRI fusion minimizes radiation dosage, patients’ discomfort, as well as the time necessary for diagnosis. These benefits subsequently make this combined imaging modality a progressive interventional tool in neuroimage analysis and diagnosis, calling for more availability despite current limitations concerning cost and access to the neuroimaging product.

In conclusion, the possibility of the simultaneous use of PET in combination with MRI in a single combination is critically important for the development of new methods to study the intricacies of various brain diseases. This will open up further possibilities for creating more effective equipment and examination techniques. Future exploration and utilization of the discussed integrative approaches will enhance the fields of neuroscience and neurology, leading to the dissemination of positive health results for all populations.
